# Adverse Drug Reactions Associated with Concomitant Use of Calcium Channel Blockers and Cocaine: An Analysis of FDA Adverse Events Reporting System Data

**DOI:** 10.3390/jcm14103461

**Published:** 2025-05-15

**Authors:** Stefania Chiappini, Alessio Mosca, Duccio G. Papanti Pelletier, John M. Corkery, Amira Guirguis, Davide Arillotta, Giovanni Martinotti, Fabrizio Schifano

**Affiliations:** 1 Faculty of Medicine, Department of Psychiatry, UniCamillus University of Medical Sciences, Via di S. Alessandro 8, 00131 Rome, Italy; 2 Department of Neurosciences, Imaging, and Clinical Sciences, University of Chieti-Pescara, 66100 Chieti, Italygiovanni.martinotti@gmail.com (G.M.); 3 Cividale Community Mental Health Centre, ASUFC Mental Health Department, Friuli Venezia Giulia, 33100 Udine, Italy; ducciopapanti@gmail.com; 4 Psychopharmacology, Drug Misuse, and Novel Psychoactive Substances Research Unit, University of Hertfordshire, Hertfordshire AL10 9AB, UK; j.corkery@herts.ac.uk (J.M.C.); davide.arillotta@yahoo.it (D.A.); f.schifano@herts.ac.uk (F.S.); 5 Pharmacy, Swansea University Medical School, Swansea University, Wales SA28PP, UK; amira.guirguis@swansea.ac.uk; 6 Department of Pharmacology and Toxicology, University of Florence Medical School, 50134 Firenze, Italy

**Keywords:** calcium channel blockers, cocaine, stimulants, drug misuse, recreational drug abuse, drug mortality

## Abstract

**Background**: Recent drug enforcement activities have possibly suggested the presence of some calcium channel blocker antihypertensives in association with cocaine. The seizure revealed the possibility that the two drugs might have been used together for some unknown reasons. **Methods**: Hence, this study aimed at investigating the nature and frequency of adverse drug reactions (ADRs) reported in association with the concomitant use of calcium channel blockers (CCBs) and cocaine, using data from the FDA Adverse Event Reporting System (FAERS). **Results**: After removing duplicate reports, a total of 67 cases involving concomitant use of cocaine and CCBs were analyzed and were stratified into three groups based on the CCB involved: verapamil (n = 19), diltiazem (n = 30), and amlodipine (n = 18). Logistic regression analysis identified “product use for unknown indication” (β = 0.33) as the strongest predictor of fatal outcomes. Age revealed a modest negative association with fatal outcome (β = −0.93, intercept = 4.07). Concomitant substance use was reported in over 84% of cases. Frequently co-used substances included opioids, benzodiazepines, antidepressants, antihistamines, and alcohol. Poly-drug use was most pronounced in the diltiazem group, which exhibited the highest burden of opioid and alcohol co-exposure. **Conclusions**: It is here suggested that clinicians should exercise caution when managing individuals who use cocaine, due to the potential for increased toxicity and lethality when CCBs are co-used, either as part of a prescribed treatment or if CCBs are present as adulterants in cocaine.

## 1. Introduction

In late February 2025, law enforcement authorities in Cuneo, Italy, conducted a search of a residence occupied by two foreign nationals, resulting in their arrest on charges of drug possession with intent to supply [1]. The operation led to the seizure of significant quantities of hashish and crack cocaine. Notably, officers also discovered 12 packages of nifedipine, a prescription antihypertensive medication [1]. The seizure revealed the possibility that cocaine and the antihypertensive drugs might have been used together for reasons that still need to be clarified. Indeed, cocaine exerts its central effects primarily by blocking the reuptake of norepinephrine and dopamine at key neurotransmission sites, leading to excessive catecholaminergic stimulation [2,3]. Additionally, serotonergic pathways have been implicated in the neurotoxicity associated with cocaine use [4]. The heightened catecholamine activity within various central nervous system (CNS) circuits is believed to underlie the characteristic symptoms of euphoria, agitation, and hallucinations [2]. In contrast, the mechanisms behind its cardiovascular toxicity are less clearly defined. However, it is postulated that cocaine enhances adrenergic signaling by increasing sympathetic nervous system activity, resulting in tachycardia, hypertension, and focal vasoconstriction [5]. The adulteration of illicit substances, particularly cocaine, with various pharmacological agents poses significant health risks to users. While the incorporation of local anesthetics, amphetamines, and other compounds as adulterants is well-documented [6], the specific use of antihypertensive drugs for this purpose is less clearly established in the recent literature. Notably, xylazine, a veterinary sedative with antihypertensive properties, has been identified as a frequent adulterant in illicit drug markets, especially in regions like Puerto Rico and the northeastern United States [7]. Xylazine’s combination with opioids, colloquially termed “tranq dope”, has raised concerns due to its severe CNS depressant effects and potential for profound hypotension [8]. However, comprehensive studies detailing the deliberate inclusion of other antihypertensive medications as cocaine adulterants remain limited.

### Aim of the Study

This study aims to investigate the nature and frequency of adverse drug reactions (ADRs) reported in association with the concomitant use of calcium channel blockers (CCBs) and cocaine, using data from the Food and Drug Administration Adverse Event Reporting System (FAERS).

## 2. Methods

### 2.1. Study Design and Data Collection

For this study, ADRs were extracted from the FAERS as of 4 March 2024, with a specific focus on the following CCB medications: nimodipine, felodipine, isradipine, nicardipine, nisoldipine, verapamil, diltiazem, and amlodipine. The FAERS database was queried using standardized (generic) drug names to ensure consistency. Advanced text mining and natural language processing (NLP) techniques were applied to automatically identify and extract drug names from the free-text fields in the FAERS reports. Data cleaning and standardization were conducted to correct misspellings, abbreviations, and variations in drug names, including brand names, to maintain consistency across the dataset. Data were finally extracted from structured reports and a compiled dataset, including patient demographics, clinical outcomes, ADRs, country of origin, reporter type, and the presence of concomitant drugs. Due to the aggregated nature of part of the dataset, quantitative variables were manually structured into categorical summaries using Python (pandas library). A retrospective analysis was conducted on 67 documented cases of concomitant use of cocaine and CCBs, subdivided into three groups based on the specific CCB involved: verapamil (n = 19), diltiazem (n = 30), and amlodipine (n = 18).

### 2.2. Descriptive and Exploratory Analyses

Descriptive statistics were used to evaluate the distribution of age, sex, geographic origin, ADR types, clinical outcomes, and patterns of concomitant drug use across the three CCB subgroups. Age was categorized into four groups: 0–25, 26–40, 41–65, and >65 years. Frequencies and proportions were reported for each variable. Data visualization, including bar plots, was performed using seaborn and matplotlib libraries in Python 3.11.

### 2.3. Correlation and Predictive Modeling

To explore potential associations between variables, Pearson correlation coefficients were calculated in Microsoft Excel for Microsoft 365 (version 2402) to investigate associations among quantitative and ordinal variables, including age distributions, fatality rates, and classes of concomitant drugs. A correlation matrix was generated to highlight possible predictors of fatality. A binary logistic regression model was implemented to assess predictors of fatal outcomes. In one model, age (standardized using StandardScaler) was used as the independent variable, and fatal outcome as the binary dependent variable. Due to aggregated data, a synthetic micro-dataset was created by decomposing group counts into individual entries with midpoint age values and probabilistically assigned outcomes based on observed fatality rates. In a second logistic regression model, ADR categories and counts of concomitant drug classes (e.g., opioids, benzodiazepines) were included as predictors. Model coefficients were reported to assess relative feature importance. The category “completed suicide” was excluded as a predictor, as it constitutes a fatal outcome by definition.

### 2.4. Dimensionality Reduction and Clustering

Principal Component Analysis (PCA) was conducted using Python 3.11.5 (64-bit, USA origin) with the scikit-learn library (version 1.3.2) to reduce dimensionality and visually assess clustering of cases based on the complete ADR and drug profile. Additionally, unsupervised K-means clustering (k = 2) was performed using scikit-learn, and data visualization was implemented with matplotlib and seaborn libraries to identify and illustrate data-driven groupings based on pharmacological and clinical features.

### 2.5. Ethical Considerations

As the data were reported anonymously, ethical approval was not required.

## 3. Results

Among the CCBs analyzed, no ADRs were reported in association with cocaine use for nimodipine, felodipine, isradipine, nicardipine, or nisoldipine. However, ADRs were identified for verapamil (71 cases), diltiazem (103 cases), and amlodipine (74 cases) in the context of concomitant cocaine use over the 2000–2024 timeframe. After removing duplicate reports, a total of 67 cases involving concomitant use of cocaine and CCBs were analyzed, and were stratified into three groups based on the CCB involved: verapamil (n = 19), diltiazem (n = 30), and amlodipine (n = 18).

**Figure 1:**
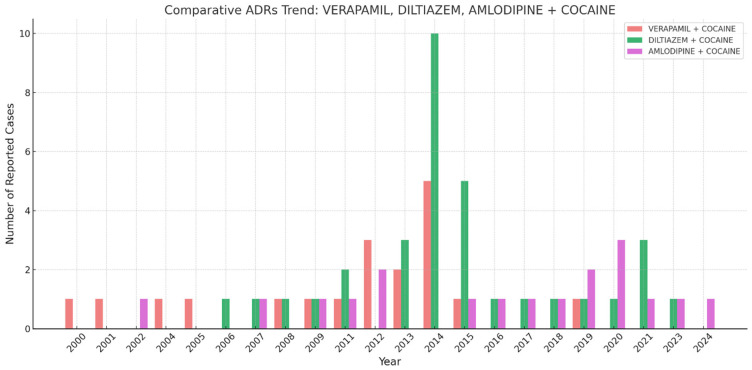
Comparative adverse drug reaction trends for concomitant use of cocaine with verapamil, diltiazem, and amlodipine.

Figure 1 describes the number of reported ADRs over time for the three CCBs, verapamil, diltiazem, and amlodipine, in combination with cocaine. The highest spike appears around 2014, where ‘Diltiazem + Cocaine’ showed a peak of 10 cases. Verapamil and amlodipine presented with fluctuating but generally lower numbers of reported cases compared to diltiazem. The number of reports remained relatively low before 2010, increased around 2012–2015, and then fluctuated with smaller peaks in the 2020s.

Most individuals were aged between 41 and 65 years, accounting for 57.9%, 33.3%, and 38.9% of cases in the verapamil, diltiazem, and amlodipine groups, respectively. The second most common age group was 26–40 years. A male predominance was observed in the verapamil (10/15) and diltiazem (21/25) groups, while the amlodipine group had a slight female predominance (8/13). Approximately 26–28% of cases lacked reported sex data (Table 1). Most cases were reported in the United States (n = 43), followed by Germany (n = 5), France (n = 1), and South Africa (n = 2). Most reports came from healthcare professionals (n = 58), with limited consumer submissions and some unspecified sources. Also, CCBs were recorded as prescribed for unknown indications.

**Figure 2:**
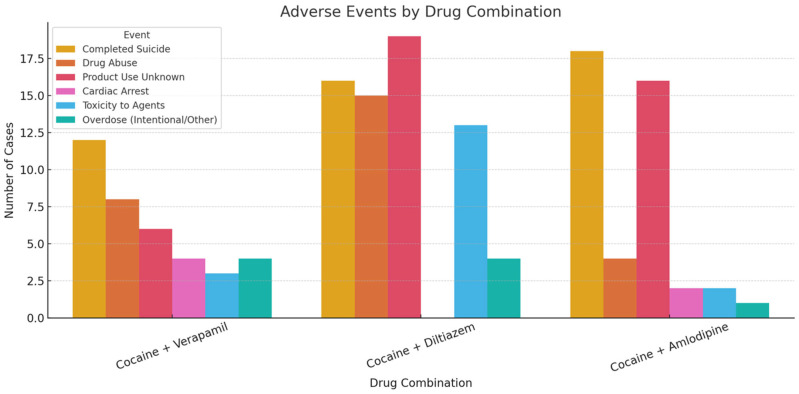
Fatal cases reported by drug combination.

Fatal outcomes were observed in 18/19 (94.7%) of verapamil cases, 29/30 (96.7%) of diltiazem cases, and 18/18 (100%) of amlodipine cases (Figure 2).

“Completed suicide” emerged as the most frequently reported adverse drug reaction, particularly in the amlodipine group (100%), while other outcomes such as cardiac arrest and respiratory arrest were reported less frequently. Logistic regression analysis identified “product use for unknown indication” (β = 0.33) as the strongest predictors of fatal outcomes. Descriptive analysis revealed that “completed suicide” was the most commonly reported fatal adverse drug reaction across all groups. Therefore, it was not included as a predictor in the logistic regression model for fatal outcomes, as it constitutes a fatal outcome by definition. Another logistic regression model using age as a predictor revealed a modest negative association with fatal outcome (β = −0.93, intercept = 4.07), with an overall classification accuracy of 97.7%, although model interpretation was limited due to class imbalance.

Details on non-fatal cases are reported in Table 2.

**Figure 3:**
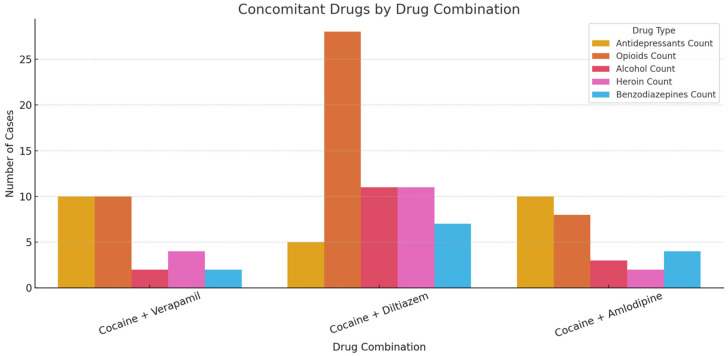
Concomitant drugs by drug combination.

Concomitant drug use was reported in over 84% of cases. Frequently co-used substances included opioids (e.g., fentanyl, tramadol, codeine), benzodiazepines, antidepressants (e.g., venlafaxine, citalopram), antihistamines, and alcohol. Poly-drug use was most pronounced in the diltiazem group, which exhibited the highest burden of opioid and alcohol co-exposure (Figure 3).

Table 3 specifically reports information regarding cases where CCBs and cocaine co-use occurred without additional substances.

Unsupervised K-means clustering (k = 2) identified two distinct case profiles:

- One cluster was characterized by high rates of opioid and alcohol co-use.
- The second cluster was dominated by suicide-related ADRs.

PCA showed partial separation of the drug groups, with diltiazem cases demonstrating the most complex pharmacological profiles.

## 4. Discussion

The current study represents the first attempt to study the interaction between CCBs and cocaine using pharmacovigilance data. Our results indicated that the majority of cases originated from the United States. This finding is somehow not surprising, since the United States presents with a high prevalence level of both drug abuse [15] and ‘pharming’ [16] practices. It is important to note, however, that the United States is among those few regions with established adverse event reporting systems, such as the FAERS [17].

Whilst CCBs were hypothesized to protect against the toxic effects of cocaine, specifically the development of dependence [18] and its adrenergic/stimulant-induced hypertension [19], preclinical studies demonstrated instead potentiation of the toxic effects of cocaine [20]. Indeed, animal studies have shown that the combination of cocaine and CCBs such as diltiazem, nifedipine, or verapamil can accelerate the onset of seizures and increase mortality episodes, which can occur in a few minutes [21]. Several mechanisms by which CCBs may increase the cocaine-associated toxicity levels have been reported: (i) vasodilation induced by CCBs may lead to enhanced delivery of cocaine to cerebral tissues; (ii) CCBs may increase cocaine toxicity by interacting at selected CNS sites; and (iii) whilst affecting cell membrane function, adenosine activity, neurotransmitter synthesis, and subsequent neurotransmitter release, CCBs could potentiate the reuptake blockade of neuroamines induced by cocaine in the synaptic cleft, enhancing the effect of cocaine itself [22]. Non-dihydropyridine CCBs, and particularly verapamil and diltiazem, may be associated with increased lethality when co-used with cocaine. This is likely due to both synergistic pharmacodynamic effects and CYP3A4-mediated drug interactions that enhance toxicity and complicate clinical management [23]. In this context, current clinical guidelines recommend that CCBs should not be used as first-line treatment for cocaine-associated chest pain. However, their use may be considered in patients who do not respond to benzodiazepines and nitro-glycerine [24].

Considering the cardiovascular risks of cocaine and crack cocaine, which include acute hypertension, tachycardia, vasospasm leading to myocardial infarction, arrhythmias, stroke, and related fatalities [25,26,27], one could wonder if the intentional adulteration of cocaine with CCBs was carried out to mitigate these effects in people who use cocaine chronically or acutely. Indeed, the licensed therapeutic indications of verapamil include the treatment of hypertension and the secondary prevention of re-infarction after an acute myocardial infarction in patients without heart failure who are not receiving diuretics and for whom beta-blockers are not appropriate [28]. Conversely, diltiazem is indicated for the management of angina pectoris and the treatment of mild to moderate hypertension [29].

Conversely, the uncontrolled use of CCBs as adulterants poses severe health risks, including the potential for profound hypotension and cardiovascular complications. Conversely, it is here tentatively suggested that clients taking cocaine on a regular basis suffer from hypertension, and as a result, they may be frequently considered for a CCB prescription; as a result, they were here spuriously identified as CCBs + cocaine misusers.

Logistic regression analysis identified “product use for unknown indication” as the strongest predictor of fatal outcomes. This finding appears to support the notion that the misuse of medications taken without medical prescription may pose a significant health risk (Chiappini & Schifano, 2020) [16,30].

Another interesting point is the current study’s high prevalence of fatalities, and specifically of ADRs classified as suicides, which might show the severe effects deriving from the interaction between CCBs and cocaine. Unfortunately, due to data limitations, it was not possible here to ascertain which were the drug dosages being prescribed and the medical diagnoses given in the cases considered. However, most cases were on antidepressants drugs and opioids, hence suggesting a depression diagnosis or an opioid use disorder.

This study is the first to explore the potential interactions between CCBs and cocaine using pharmacovigilance data. Contrary to earlier hypotheses suggesting a protective role of CCBs, current findings may instead be in line with preclinical evidence indicating that CCBs may potentiate the toxic effects of cocaine. In particular, non-dihydropyridine CCBs, especially verapamil and diltiazem, were here associated with increased lethality levels when used in combination with cocaine.

### Limitations

Whilst the findings of this study seem to be both relevant and clinically significant, several limitations must be acknowledged. First, a thorough pharmacovigilance analysis, encompassing metrics such as the reporting odds ratio (ROR), proportional reporting ratio (PRR), information component (IC), and Bayesian empirical geometric mean (EBGM), could not be conducted here due to the relatively small number of cases involved. Second, the analysis was further constrained by limited data on patient demographics, medical histories, and specific drug formulations, restricting the ability to explore the role of risk factors or potential causal links in greater detail. Additionally, evaluating ADRs in isolation is rarely sufficient to establish causality, as observed outcomes may be influenced by the underlying condition, the development of a new health issue, or drug interactions. Here, events reported occurred in the context of concomitant substance use (e.g., cocaine); thus, they might not be directly classified as ADRs without a clear, causality-assessed link to the medicine. Finally, the frequency of case reports related to a specific drug or suspected ADR can be influenced not only by the actual occurrence of the event but also by factors such as drug usage patterns, the characteristics of the reaction, public awareness, and variability in reporting practices, factors that may contribute to underreporting and underestimation of the true incidence and severity of ADRs.

## 5. Conclusions

Clinicians should exercise caution when managing individuals who use cocaine, due to the potential for increased toxicity and lethality when CCBs are co-used, either as part of a prescribed treatment or if CCBs are present as adulterants in cocaine. Alternative treatment options should be considered for cardiovascular conditions in these patients, and close monitoring is advised for signs of cardiovascular and neurological complications.

## Figures and Tables

**Table 1:** Data regarding retrieved cases involving CCBs and cocaine (source: FAERS 2000–2024).

	Cocaine + Verapamil (Tot. Cases: 19)	Cocaine + Diltiazem (Tot. Cases: 30)	Cocaine + Amlodipine (Tot. Cases: 18)
AGE (years)	0–25: 0 26–40: 1 41–65: 11 >65: 1 Not Specified: 5	0–25: 2 26–40: 4 41–65: 10 >65: 2 Not Specified: 12	0–25: 0 26–40: 3 41–65: 7 >65: 2 Not Specified: 6
SEX (F/M)	5/10 Not Specified 4	4/21 Not Specified 5	8/5 Not Specified 5
OUTCOME	Fatal 18 Life Threatening 1	Fatal 29 Other 1	Fatal 18
ADVERSE DRUG REACTIONS	Completed suicide 12 Drug abuse 8 Product use for unknown indication 6 Cardiac arrest 4 Toxicity to various agents 3 Suicide attempt 2 Overdose/Intentional overdose 4 Respiratory arrest 2	Product use for unknown indication 19 Completed suicide 16 Drug abuse 15 Toxicity to various agents 13 Overdose 4 Intentional drug misuse 3 Homicide 1 Suspected suicide 1 Suicidal ideation 1 Accidental death 1 Poisoning 1	Completed suicide 18 Product use for unknown indication 16 Drug abuse 4 Product use issue 1 Cardiac arrest 2 Homicide 1 Toxicity to various agents 2 Overdose 1 Substance abuse 1
COUNTRY	United States 13 Germany 1 Not Specified 5	United States 15 Germany 3 France 1 South Africa 1 Not Specified 16	United States 15 South Africa 1 Germany 1 Not Specified 1
REPORTER	Healthcare professional 14 Consumer 1 Not Specified 2	Healthcare professional 26 Not Specified 4	Healthcare professional 18
CASES WITHOUT CONCOMITANT DRUGS	3	1	5
CONCOMITANT DRUGS	Antidepressant drugs (Bupropion, Citalopram, Venlafaxine, Trazodone, Amitriptyline, Mirtazapine) 10 Opioids (Fentanyl, Hydrocodone, Codeine, Methadone, Oxycodone, Tramadol) 10 Acetaminophen/Paracetamol 6 Heroin 4 Antipsychotic medications (Risperidone, Quetiapine, Doxepin): 3 Antihistamine drugs (Diphenhydramine, Promethazine) 2 Alcohol 2 Z-drugs (Zolpidem): 2 Mood stabilizers (Lamotrigine) 1 Benzodiazepines (Alprazolam) 2 Ketamine 1	Opioids (Codeine, Fentanyl, Tramadol, Methadone, Hydrocodone, Oxycodone) 28 Alcohol 11 Heroin 11 Acetaminophen/Paracetamol 8 Antihistamine drugs (Diphenhydramine, Cetirizine, Promethazine) 7 Benzodiazepines (Alprazolam, Clonazepam, Diazepam, Midazolam) 7 Antidepressant drugs (Clomipramine, Fluoxetine, Nortriptyline, Trazodone) 5 Amphetamine/Methamphetamine 4 Dextromethorphan 3 THC 3 Mood stabilizers (Gabapentin, Topiramate) 1 Pseudoephedrine 1 Antipsychotic medications (Risperidone) 1	Antidepressant drugs (Amitriptyline, Citalopram, Duloxetine, Clomipramine, Fluoxetine, Nortriptyline, Paroxetine, Trazodone, Venlafaxine) 10 Antihistamine drugs (Diphenhydramine, Promethazine) 4 Mood stabilizers (Gabapentin, Topiramate) 3 Opioids (Codeine, Fentanyl, Tramadol) 8 Benzodiazepines (Alprazolam, Clonazepam, Lorazepam) 4 Acetaminophen/Paracetamol 4 THC 3 Alcohol 3 Heroin 2 Antipsychotic medications (Quetiapine, Risperidone) 2 Pseudoephedrine 1 Amphetamine/Methamphetamine 2 Zolpidem 1

**Table 2:** Detailed information on non-fatal cases involving CCBs and cocaine (source: FAERS 2000–2024).

	Cocaine + Verapamil (1/19 Cases)	Cocaine + Diltiazem (1/30)
OUTCOME	Life-threatening	Other
ACTIVE INGREDIENTS	Cocaine; Verapamil Hydrochloride; Alcohol	Fentanyl; Hydroxyzine Hydrochloride; Tramadol; Codeine; Prazepam; Tadalafil; Morphine; Benzoylecgonine; Diamorphine; Levamisole; Methadone Hydrochloride; Amphetamine; Ketamine; Cocaine; Sildenafil; Diltiazem; Lidocaine; Amitriptyline; Etifoxine; Nortriptyline; Cetirizine Hydrochloride; Methamphetamine; Dextromethorphan
REACTIONS	Suicide Attempt; Toxicity To Various Agents; Alcohol Poisoning; Hypomagnesaemia; Hypophosphataemia; Hypotension; Drug Interaction; Lethargy; Vomiting; Opiates Positive; Hypokalaemia	Drug Abuse; Toxicity to Various Agents; Product Use for Unknown Indication
SEX	Female	Male
AGE (years)	36	42
SENDER	Recro Pharma	Johnson And Johnson
REPORTER	Healthcare Professional	Healthcare professional
COUNTRY	United States	France
Ref.	Yuan et al., 1999 [9]	-

**Table 3:** Information regarding cases where CCBs and cocaine co-use occurred WITHOUT additional substances (source: FAERS 2000–2024).

	Cocaine + Verapamil (3/19 Cases)	Cocaine + Diltiazem (1/30)	Cocaine + Amlodipine (5/18)
OUTCOME	Died (3)	Died	Died (5)
REACTIONS	Completed Suicide (3)	Completed Suicide	Completed Suicide (5) Other Outcomes (1) Toxicity to Various Agents (3) Product Used for Unknown Indication (1) Cardiac Arrest (1) Respiratory Arrest (1) Ill-Defined Disorder (1) Drug Abuser (1)
SEX	Female (1) Male (2)	Male	Male (2) Not Specified (3)
AGE (years)	50 (1), 53 (1), 65 (1)	47	29 (1), 38 (1), 52 (2), 59 (1)
SENDER	Pfizer (2) Novartis (1)	Apotex	Apotex (1) Aurobindo (1) Pfizer (2) Roxane (1)
REPORTER	Healthcare Professional (3)	Healthcare Professional	Healthcare Professional (5)
COUNTRY	United States (3)	Not Specified	United States (3) Not Specified (2)
Ref.	Bronstein et al., 2010 [10] Bronstein 2012 [11] Mowry 2013 [12]	-	Gummin et al., 2019 [13] Mowry et al., 2016 [14] -(3)

## Data Availability

The FAERS data were available through the FAERS Public Dashboard on their publicly available website: https://www.fda.gov (accessed on 4 March 2025).

## References

[1] ANSA.it Con la Droga Sequestrati Anti-Ipertensivi, Allarme a Cuneo. 2 March 2025. https://www.ansa.it/piemonte/notizie/2025/03/02/con-la-droga-sequestrati-anti-ipertensivi-allarme-a-cuneo_29d72eb9-3a22-45b0-a340-f1d6133724ba.html.

[2] Davis S., Zhu J. (2022). Substance abuse and neurotransmission. Adv. Pharmacol..

[3] Volkow N.D., Wang G.J., Telang F., Fowler J.S., Logan J., Childress A.R., Jayne M., Ma Y., Wong C. (2006). Cocaine cues and dopamine in dorsal striatum: Mechanism of craving in cocaine addiction. J. Neurosci. Off. J. Soc. Neurosci..

[4] Nestler E.J. (2005). The neurobiology of cocaine addiction. Sci. Pract. Perspect..

[5] Richards J.R., Hollander J.E., Ramoska E.A., Fareed F.N., Sand I.C., Izquierdo Gómez M.M., Lange R.A. (2017). β-Blockers, Cocaine, and the Unopposed α-Stimulation Phenomenon. J. Cardiovasc. Pharmacol. Ther..

[6] Kudlacek O., Hofmaier T., Luf A., Mayer F.P., Stockner T., Nagy C., Holy M., Freissmuth M., Schmid R., Sitte H.H. (2017). Cocaine adulteration. J. Chem. Neuroanat..

[7] Mumba M.N., Tice J., Brown W. (2023). Xylazine: The Drug Taking the World By Storm: What You Need to Know. J. Psychosoc. Nurs. Ment. Health Serv..

[8] Edinoff A.N., Sall S., Upshaw W.C., Spillers N.J., Vincik L.Y., De Witt A.S., Murnane K.S., Kaye A.M., Kaye A.D. (2024). Xylazine: A Drug Adulterant of Clinical Concern. Curr. Pain Headache Rep..

[9] Yuan T.H., Kerns W.P., Tomaszewski C.A., Ford M.D., Kline J.A., Kline J. (1999). Insulin-Glucose as Adjunctive Therapy for Severe Calcium Channel Antagonist Poisoning. J. Toxicol. Clin. Toxicol..

[10] Bronstein A.C., Spyker D.A., Cantilena L.R., Green J.L., Rumack B.H., Giffin S.L. (2010). 2009 Annual Report of the American Association Of Poison Control Centers’ National Poison Data System (NPDS): 27th Annual Report. Clin. Toxicol..

[11] Bronstein A. (2012). 2011 Annual Report of the American Association of Poison Control Centers’ National Poison Data System (NPDS): 29th Annual Report. Clin. Toxicol..

[12] Mowry J. (2013). 2012 Annual Report of the American Association of Poison Control Centers’ National Poison Data System (NPDS): 30th Annual Report. Clin. Toxicol..

[13] Gummin D.D., Mowry J.B., Spyker D.A., Brooks D.E., Beuhler M.C., Rivers L.J., Hashem H.A., Ryan M.L. (2019). 2018 Annual Report of the American Association of Poison Control Centers’ National Poison Data System (NPDS): 36th Annual Report. Clin. Toxicol..

[14] Mowry J.B., Spyker D.A., Brooks D.E., Zimmerman A., Schauben J.L. (2016). 2015 Annual Report of the American Association of Poison Control Centers’ National Poison Data System (NPDS): 33rd Annual Report. Clin. Toxicol..

[15] UNODC (2024). World Drug Report 2024 (United Nations Publication, 2024).

[16] Chiappini S., Schifano F. (2020). What about “Pharming”? Issues Regarding the Misuse of Prescription and Over-the-Counter Drugs. Brain Sci..

[17] Chiappini S., Vickers-Smith R., Guirguis A., Corkery J.M., Martinotti G., Schifano F. (2022). A Focus on Abuse/Misuse and Withdrawal Issues with Selective Serotonin Reuptake Inhibitors (SSRIs): Analysis of Both the European EMA and the US FAERS Pharmacovigilance Databases. Pharmaceuticals.

[18] Vitcheva V., Simeonova R., Karova D., Mitcheva M. (2011). Nifedipine lowers cocaine-induced brain and liver enzyme activity and cocaine urinary excretion in rats. Arh. Za Hig. Rada I Toksikol..

[19] Schindler C.W., Tella S.R., Prada J., Goldberg S.R. (1995). Calcium channel blockers antagonize some of cocaine’s cardiovascular effects, but fail to alter cocaine’s behavioral effects. J. Pharmacol. Exp. Ther..

[20] Ansah T.A., Wade L.H., Kopsombut P., Shockley D.C. (2002). Nifedipine potentiates the toxic effects of cocaine in mice. Prog. Neuro-Psychopharmacol. Biol. Psychiatry.

[21] Derlet R.W., Albertson T.E. (1989). Potentiation of cocaine toxicity with calcium channel blockers. Am. J. Emerg. Med..

[22] Leenen F.H., Ruzicka M., Huang B.S. (2001). Central sympathoinhibitory effects of calcium channel blockers. Curr. Hypertens. Rep..

[23] Yeo K.R., Yeo W.W. (2001). Inhibitory effects of verapamil and diltiazem on simvastatin metabolism in human liver microsomes. Br. J. Clin. Pharmacol..

[24] McCord J., Jneid H., Hollander J.E., de Lemos J.A., Cercek B., Hsue P., Gibler W.B., Ohman E.M., Drew B., Philippides G. (2008). Management of cocaine-associated chest pain and myocardial infarction: A scientific statement from the American Heart Association Acute Cardiac Care Committee of the Council on Clinical Cardiology. Circulation.

[25] Alawoè C., Chapet N., Roubille F., Peyrière H., Eiden C. (2024). Narrative Review of Heart Failure Related to Cocaine Consumption and Its Therapeutic Management. J. Clin. Med..

[26] Darke S., Duflou J., Peacock A., Chrzanowska A., Farrell M., Lappin J. (2023). Clinical characteristics of fatal cocaine toxicity in Australia, 2000–2021. Drug Alcohol Rev..

[27] Hsue P.Y., McManus D., Selby V., Ren X., Pillutla P., Younes N., Goldschlager N., Waters D.D. (2007). Cardiac arrest in patients who smoke crack cocaine. Am. J. Cardiol..

[28] Verapamil. https://www.medicines.org.uk/emc/product/10613/smpc.

[29] Diltiazem. https://www.medicines.org.uk/emc/product/7583/smpc.

[30] Chiappini S., Miuli A., Mosca A., Pettorruso M., Guirguis A., John M.C., Martinotti G., Di Giannantonio M., Schifano F. (2021). The Benzydamine Experience: A Systematic Review of Benzydamine Abuse. Curr. Neuropharmacol..

